# The Role of Autophagy in Gastric Cancer Chemoresistance: Friend or Foe?

**DOI:** 10.3389/fcell.2020.621428

**Published:** 2020-12-03

**Authors:** Jing-Li Xu, Li Yuan, Yan-Cheng Tang, Zhi-Yuan Xu, Han-Dong Xu, Xiang-Dong Cheng, Jiang-Jiang Qin

**Affiliations:** ^1^Institute of Cancer and Basic Medicine, Chinese Academy of Sciences, Hangzhou, China; ^2^Cancer Hospital of the University of Chinese Academy of Sciences, Zhejiang Cancer Hospital, Hangzhou, China; ^3^The First Clinical Medical College of Zhejiang Chinese Medical University, Hangzhou, China; ^4^School of Chinese Medicine, Hong Kong Baptist University, Kowloon Tsai, Hong Kong, China

**Keywords:** gastric cancer, autophagy, chemoresistance, ncRNAs, natural products, inhibitor and activator

## Abstract

Gastric cancer is the third most common cause of cancer-related death worldwide. Drug resistance is the main inevitable and vital factor leading to a low 5-year survival rate for patients with gastric cancer. Autophagy, as a highly conserved homeostatic pathway, is mainly regulated by different proteins and non-coding RNAs (ncRNAs) and plays dual roles in drug resistance of gastric cancer. Thus, targeting key regulatory nodes in the process of autophagy by small molecule inhibitors or activators has become one of the most promising strategies for the treatment of gastric cancer in recent years. In this review, we provide a systematic summary focusing on the relationship between autophagy and chemotherapy resistance in gastric cancer. We comprehensively discuss the roles and molecular mechanisms of multiple proteins and the emerging ncRNAs including miRNAs and lncRNAs in the regulation of autophagy pathways and gastric cancer chemoresistance. We also summarize the regulatory effects of autophagy inhibitor and activators on gastric cancer chemoresistance. Understanding the vital roles of autophagy in gastric cancer chemoresistance will provide novel opportunities to develop promising therapeutic strategies for gastric cancer.

## Introduction

Gastric cancer is one of the most common gastrointestinal tumors in the world, with more than one million new cases every year, and remains the third leading cause of cancer-related deaths (Fitzmaurice et al., 2019; Thrift and El-Serag, 2019). For patients with early gastric cancer, surgical resection is the best treatment option. However, more than 60% of patients have developed local or distant metastasis at diagnosis, which causes that most of the patients do not have the opportunity to receive surgical treatment (Yuan et al., 2020). Hence, chemotherapy-based comprehensive treatment is the main choice for most patients with middle- and late-stage gastric cancer (Zhang et al., 2020). However, poor or even no response to chemotherapy is frequently observed in the treatment of gastric cancer patients due to the intrinsic or acquired drug resistance, which becomes the most detrimental cause of treatment failure and low survival rate (Biagioni et al., 2019).

Autophagy, as a major intracellular degradation system in eukaryotic cells that degrades and clears defective or aging organelles, can be divided into three major forms according to the different mechanisms of clearing intracellular components: macroautophagy, microautophagy, and chaperone-mediated autophagy (Galluzzi and Green, 2019). Among them, macroautophagy (hereafter autophagy) is the most common and intensively studied form (Amaravadi et al., 2019; Abdrakhmanov et al., 2020). Previous studies have shown that autophagy plays a double-edged sword role at different stages of tumorigenesis. At the early stage, autophagy plays a “tumor-suppressor” function, which can stabilize the genome, protect the damaged tissues and cells, and suppress tumor occurrence, proliferation, invasion, and metastasis (Høyer-Hansen and Jäättelä, 2008). On the contrary, once the tumor is formed, autophagy plays an oncogenic function that provides cancer cells with needed survival contexts, such as nutrition to resist stress (especially after chemotherapy treatment) (Kimmelman and White, 2017). The specific roles of autophagy in tumors depend on tumor type and tumor heterogeneity, which is regulated by multiple proteins and non-coding RNAs (ncRNAs) (Jiang et al., 2020; Perez-Montoyo, 2020).

Traditional medicine (TM) has been widely used in China, South Korea, and Japan for thousands of years to treat various diseases, usually in the form of decoctions with 2–15 kinds of herbs (Qin et al., 2017; Hempen and Hummelsberger, 2020). Numerous TM decoctions or natural products derived from medical herbs have been proven to have anticancer activities (Li et al., 2013; Qin et al., 2013; Yang et al., 2019; Yuan et al., 2019). Also, dietary natural products have been shown to regulate autophagy and thus enhance the chemosensitivity of cancer cells (Emanuele et al., 2018; Dutta et al., 2019). Some antibiotics, anti-inflammatory drugs, anesthetics, etc. have been shown to regulate autophagy to exert anticancer effects (Zhu et al., 2013; Jiang et al., 2019; Moon et al., 2019; Petroni et al., 2020).

The roles of autophagy in gastric cancer progression, metastasis, and prognosis have been extensively discussed in several recent reviews (Qian and Yang, 2016; Cao et al., 2019; Zhang F. et al., 2019). Herein, we provide a review focusing on the relationship between autophagy and chemotherapy resistance in gastric cancer. We comprehensively discuss the roles and molecular mechanisms of multiple proteins and newly identified ncRNAs including miRNAs and long non-coding RNAs (lncRNAs) in autophagy and gastric cancer chemotherapy resistance, as well as their potential as therapeutic targets for gastric cancer precise medicine. We also summarize the natural products and other drugs that exhibit regulatory effects on autophagy and chemotherapy resistance. Summarizing the process of autophagy in detail and classifying the different roles of autophagy in gastric chemoresistance will provide a comprehensive understanding of autophagy and develop promising therapeutic strategies for gastric cancer.

## Overview of Autophagy

Autophagy is a common, complex, physiological and pathological, and highly conserved self-metabolic process (Galluzzi and Green, 2019). During certain extreme conditions like starvation, hypoxia, or other environmental stresses, autophagy is also an indispensable process to provide energy for cell regeneration and cell survival, which functions as a cytoprotective mechanism. However, autophagic cell death, also called type II-programmed cell death, may be accompanied by excessive autophagy (Qi et al., 2020). Here, we summarize the latest updates on the process and related signaling pathways of autophagy.

### Process of Autophagy

**FIGURE 1:**
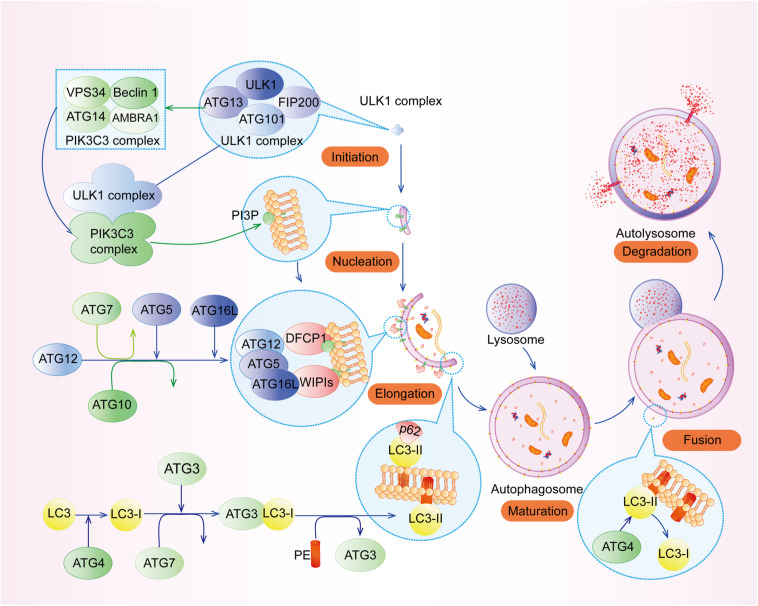
The process of autophagy. The process of autophagy can be divided into the following stages: (1) initiation, (2) vesicle nucleation, (3) vesicle elongation and maturation, (4) vesicle fusion, and (5) cargo degradation.

The process of autophagy can be divided into the following stages (Figure 1): (1) initiation, (2) vesicle nucleation, (3) vesicle elongation and maturation, (4) vesicle fusion, and (5) cargo degradation (Levy et al., 2017). Each stage of autophagy is regulated by a variety of autophagy-related genes (ATGs) such as ATG5, ATG7, ATG12, ATG16L1, and their complexes, etc. (Levine and Kroemer, 2019).

#### Initiation and Nucleation

**FIGURE 2:**
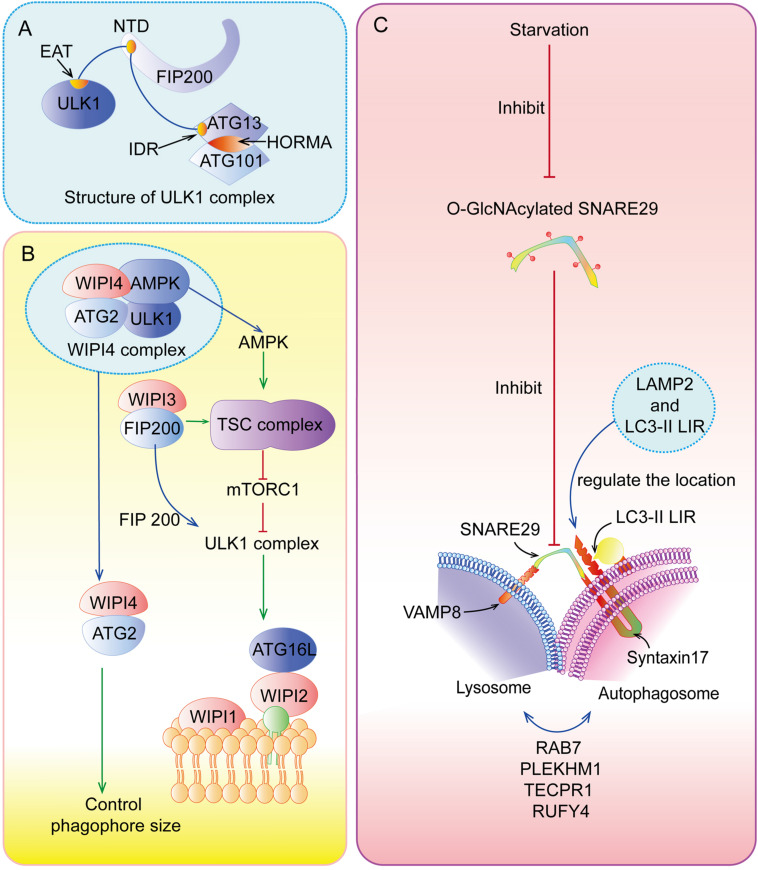
Special parts of autophagy. **(A)** The structure of ULK1 complex. **(B)** The functions of WIPIs in autophagy. **(C)** The fusion between lysosome and autolysosome.

Upon nutrient deficiency, hypoxia, and inflammation, autophagy is initiated by the unc-51-like kinase 1 (ULK1) complex (Figure 2A), composing of ULK1, FIP200 (FAK family kinase-interacting protein of 200 kDa, also known as ATG17), ATG13, and ATG101. Specifically, the intrinsically disordered region (IDR) of ATG13 interacts with ULK1 and FIP200 to form a pre-autophagosomal structure (PAS) (Hollenstein and Kraft, 2020), while its HORMA (Hop1, Rev7, and MAD2) domain forms a dimer with ATG101’s HORMA domain (Kim B. W. et al., 2018). In addition, the N-terminal 640 residues (NTD) of FIP200, shaping like a letter C with the presence of ATG13, has an intimate interaction with C-terminal IDR of ATG13 and C-terminal early autophagy targeting/tethering (EAT) domain of ULK1, ensuring the successful initiation of autophagy (Shi et al., 2020). Following the activation of ULK1 complex, the class III phosphatidylinositol 3-kinase (PI3KC3) complex, which consists of vacuolar protein sorting 34 (VPS34, also known as PI3KC3), ATG14, the activating molecule in BECN1-regulated autophagy protein 1 (AMBRA1), and the scaffold protein Beclin-1, generates phosphatidylinositol 3-phosphate (PI3P) at an endoplasmic reticulum (ER) subdomain named omegasome (Young et al., 2019; Sanchez-Martin et al., 2020). Subsequently, PI3P recruits certain effector proteins, including WIPIs (WD repeat domain phosphoinositide-interacting proteins) (Figure 2B), which can bind ATG16L1 (Bakula et al., 2017).

#### Elongation and Maturation

ATG5-ATG12 complex interacts with ATG16L1 to form the ATG12-ATG5-ATG16L1 complex, the crucial element for autophagosome elongation (Lystad et al., 2019). ATG12-ATG5-ATG16L1 complex cooperating with ATG7 and ATG3 recruits LC3 (microtubule-associated protein 1 light chain 3 alpha) to lipid phosphatidylethanolamine (PE) (Lystad et al., 2019). This type of LC3 is called LC3-II, which can elongate and close membranes in the outer surfaces of the autophagosome membrane and bind with cargo receptors such as p62 to select proteins or organelles (Zhang X. et al., 2019). In addition, recent studies have highlighted that TRAPP III-specific proteins (TRAPPC11) are necessary for the close of isolation membranes and recruitment of WIPI4-ATG2 to isolation membranes with the presence of ATG9, perhaps due to its carboxy-terminus, while TRAPPC12 is downstream of TRAPPC11 (Stanga et al., 2019). Interestingly, Guardia et al. (2020) have found that ATG9A (human ATG9), a homotrimer with four transmembrane helices, interacts with ATG2 at C-terminal platform domain of ATG9A (1–723 construct and 1-522 construct) to transfer lipids from ATG2 to ATG9A, and then ATG9A can induce the autophagosome membrane curvature for the further processes of autophagy.

#### Fusion and Degradation

After the formation and maturation of the autophagosome, most of the proteins in the outer membrane are dissociated, including ATG proteins. Then, the autophagosome fuses with the lysosome (Figure 2C) to form autolysosome in the perinuclear region (Lőrincz and Juhász, 2020). In this special area, SNAREs (soluble *N*-ethylmaleimide-sensitive factor attachment protein receptors), Syntaxin17, and SNAP29 located at autolysosome and VAMP8 located at lysosome play indispensable roles in this process (Diao et al., 2015). The localization of Syntaxin17-SNAP29 complex is regulated by vesicle-associated membrane protein 2 (LAMP2), while localization of Syntaxin17 is also regulated by LC3 due to its LC3-interacting region (LIR) (Corona and Jackson, 2018). However, recent research has found that LAMP2 can regulate this fusion without the function of Syntaxin17 in human cardiomyocytes (Chi et al., 2019). There are many other regulators of autolysosome fusion, including RAB7, NRBF2, PLEKHM1, TECPR1, RUFY4, and so on (Reggiori and Ungermann, 2017; Cai et al., 2020). At the same time, LC3-II located on outer surfaces of the membrane can be disconnected from PE by ATG4 for recycling, but internal LC3-II is degraded with cargoes and p62 by lysosomal enzymes (Nakayama et al., 2017). Finally, products of decomposition can be recycled and provided for cell growth.

### Signal Pathways Regulating Autophagy

**FIGURE 3:**
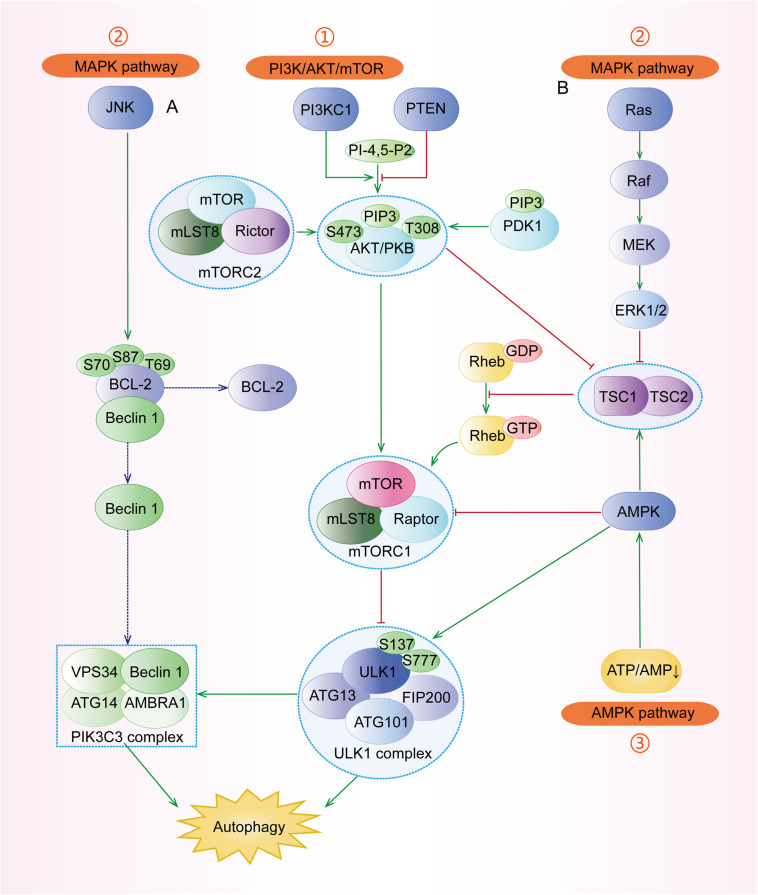
The major signaling pathways involved in the regulation of autophagy. (1) PI3K/AKT pathway: under stresses, the activated PI3KC1 makes PI-4,5-P2 into PIP3 and then activates AKT with the help of PDK1. The activated AKT, directly and indirectly, activates mTORC1 (composed of mTOR, mLST8, and Raptor) to inhibit ULK1 complex and autophagy. Specifically, the indirect pathway includes the TSC1/TSC2 complex and Rheb-GTP. (2) MAPK pathway: the major regulators include the JNK pathway and the ERK pathway. **(A)** JNK can separate the conjunction of Beclin-1 and BCL-2 by phosphorylating BCL-2 at Thr69, Ser70, and Ser87, and then Beclin-1 participates in the assembly of PIK3C3 complex to induce autophagy. **(B)** ERK has dual roles in autophagy. The successively activated Ras-Raf-MEK-ERK1/2 can directly induce autophagy. Besides, the activated ERK1/2 can block the TSC1/TSC2 complex and regulate its downstream to inhibit autophagy. (3) AMPK pathway: Under some conditions with the increasing AMP/ATP ratio, the activated AMPK promotes the formation of the TSC1/TSC2 complex to inactivate mTORC1 and regulates its downstream regulators. AMPK can also phosphorylate ULK1 at Ser317, Ser777, S467, S555, S574, and S637 to stimulate autophagy.

**FIGURE 4:**
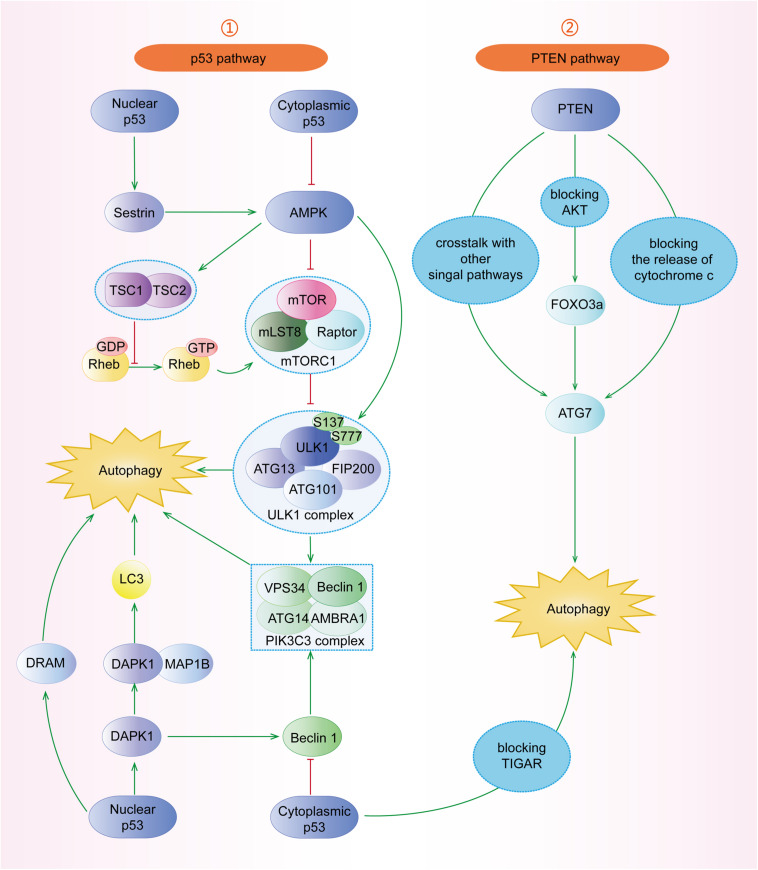
The roles of p53 and PTEN signaling pathways in the regulation of autophagy. (1) p53 pathway: nuclear p53 can induce autophagy through Sestrin1/Sestrin2-AMPK-TSC1/TSC2 complex-mTOR. DRAM is a downstream target of p53 facilitating autophagy. Furthermore, p53 can induce DAPK1 to phosphorylate Beclin-1 or bind to MAP1B to inhibit autophagy. However, cytoplasmic p53 blocks AMPK, TIGAR, and Beclin-1 to inhibit autophagy. (2) PTEN pathway: PTEN has crosstalk with other signal pathways. PTEN can induce autophagy through the PTEN/AKT/FOXO3a/Atg7 pathway, independent of mTOR and Beclin-1. Additionally, PTEN blocks the release of cytochrome c to induce autophagy.

Mammalian target of rapamycin (mTOR) complex 1 (mTORC1, made up of mTOR, Raptor, Deptor, mLST8, and PRAS40) and mTOR complex 2 (mTORC2, composed of mTOR, Rictor, Deptor, mLST8, Sin1, and PRA5/Protor-1) have a common central kinase, mTOR, a conserved serine/threonine protein kinase, which plays important roles in multiple biological processes involving in cell growth, such as regulating autophagy (Murugan, 2019). Previous studies have shown that multiple signaling pathways, such as PI3K/AKT, MAPK (mitogen activated kinase-like protein), AMPK (protein kinase AMP-activated catalytic subunit alpha 1, also known as PRKAA1) (Figure 3), p53, and PTEN (phosphatase and tensin homolog) pathways, can regulate mTORC1 and autophagy (Figure 4).

#### PI3K/AKT Pathway

The pro-oncogenic class-I PI3K (PI3KC1) can catalyze the phosphorylation of phosphatidylinositol-4,5-bisphosphate (PI-4,5-P2) into phosphatidylinositol-3,4,5-trisphosphate (PIP3), which binds both AKT and phosphoinositide-dependent protein kinase 1 (PDK1), resulting in the AKT phosphorylation at Thr308 by PDK1 (Parikh et al., 2012). The complete activation of AKT, in turn, activates its downstream mTORC1, contributing to the suppression of autophagy (Figure 3). In addition, phosphorylated AKT can phosphorylate the Ser939 and Thr1462 residues of TSC2 to block the formation of TSC1 (TSC complex subunit 1)/TSC2 complex, an inhibitor of Ras homolog enriched in the brain (Rheb). TSC1/TSC2 complex acts as a guanosine triphosphatase (GTPase)-activating protein (GAP), which can promote Rheb-GTP into Rheb-GDP; the former can activate mTORC1 while the latter cannot (Fawal et al., 2015; Yu et al., 2017). Besides, AKT can also be phosphorylated by mTORC2 at Ser473 and then the activated AKT promotes its downstream regulator, causing the inhibition of autophagy (Li et al., 2018).

#### MAPK Pathway

MAPKs play indispensable roles in many cellular processes, including cell growth, proliferation as well as autophagy (Anerillas et al., 2020). There are many subtypes of MAPKs whose upstream regulators are different, including mitogen-activated protein 3 kinases (MAPKKKs) and mitogen-activated protein 2 kinases (MAPKKs) (Gomez-Osuna et al., 2020). c-Jun N-terminal kinase (JNK) and extracellular signal-regulated kinase (ERK) are two classical and important types of MAPKs (Anerillas et al., 2020). Under various stresses (Figure 3), JNK can phosphorylate and activate BCL-2, leading to the dissociation of Beclin-1, a crucial member of PI3KC3, from BCL-2 and the subsequent induction of autophagy (Fan et al., 2016; Cheng et al., 2019). On one hand, the activated ERK1 and ERK2 can induce autophagy through Ras-Raf (MAPKKK)-MEK (MAPKK)-ERK signal pathway (Panda et al., 2015). On the other hand, the phosphorylated ERK1/2 can block the TSC1/TSC2 complex, leading to the increased expression of Rheb-GTPase to induce mTORC1, which can result in the inhibition of autophagy (Pattingre et al., 2003; Ranek et al., 2019).

#### AMPK Pathway

AMPK pathway is a key pathway to preserve energy balance and coordinate metabolism in eukaryotic cells, which is extensively involved in the regulation of autophagy, apoptosis, epithelial-mesenchymal transition (EMT), etc. (Carling, 2017; Zhang et al., 2018). Under the depletion of nutrients and energy with the increasing AMP/ATP ratio, LKB1 (liver kinase B1), a tumor suppressor kinase, stimulates the activation of AMPK, which subsequently promotes the formation of TSC1/TSC2 complex to inactivate mTORC1 and induce autophagy (Figure 3) (Bakula et al., 2017). Moreover, AMPK can inhibit mTORC1 by directly phosphorylating Raptor (Barroso-Chinea et al., 2020). Under the undernourished condition, AMPK can directly phosphorylate ULK1 at Ser317, S467, S555, S574, S637, and Ser777 by separating mTORC1 from ULK1 and stimulate autophagy (Zachari and Ganley, 2017), whereas mTORC1 can block the function of AMPK by phosphorylating ULK1 under the glucose abundant condition (Mao and Klionsky, 2011).

#### P53 Pathway

The p53 tumor suppressor participates in multiple cellular biological processes, including cell cycle arrest, apoptosis, senescence, proliferation, DNA repair, and autophagy (Qin et al., 2018; Hafner et al., 2019). However, the mechanisms of nuclear p53 and cytoplasmic p53 are different. With the expression of nuclear p53 stimulated by starvation conditions, the activated AMPK induces autophagy through the TSC1/TSC2 complex and mTOR pathway (Figure 4) (Hu et al., 2019; Gao et al., 2020). Chollat-Namy et al. (2019) have found that p53 upregulates the expression of its downstream targets Sestrin-1 and Sestrin-2, which further activate AMPK through phosphorylating AMPK at Thr172, subsequently leading to the dephosphorylation of mTORC1 at S2448 and the induction of autophagy. Damage-regulated autophagy modulator (DRAM) and death-associated protein kinase 1 (DAPK1) are other important downstream targets of p53 and have been reported to induce autophagy (Crighton et al., 2006; Zhou et al., 2016; Hu et al., 2019). At the same time, the activated DAPK1 can, in turn, stimulate and stabilize p53, forming a positive feedback loop (Hu et al., 2019). In contrast, cytoplasmic p53 inhibits autophagy through blocking AMPK, TIGAR (TP53-induced glycolysis and apoptosis regulator), and Beclin-1, whose underlying mechanisms need to be further studied (Mrakovcic and Fröhlich, 2018).

#### PTEN Pathway

The tumor suppressor PTEN has been found to act as a negative endogenous regulator of the PI3K/AKT pathway, which can hydrolyze PIP3 back to PIP2, resulting in the induction of autophagy (Figure 4) (Sun et al., 1999; Gao et al., 2018). It has also been reported that PTEN can play the role of autophagy catalysator through the AKT/mTOR pathway (Chen et al., 2015; Gao et al., 2018). PTEN regulates autophagy in PTEN/AKT/FOXO3a/ATG7 axis in non-small-cell lung cancer (NSCLC) cells, independent of mTOR and Beclin-1 (Cai et al., 2018). Specifically, the overexpression of PTEN inhibits AKT activity, which leads to the increased ATG7 expression through FOXO3a (Cai et al., 2018). Chen et al. (2015) have found that the phosphorylation of PTEN at Ser113 makes PTEN located in the nucleus, resulting in the activation of the MAPK8/JNK1-MAPK9/JNK2-sestrin2-AMPK pathway to facilitate autophagy. Furthermore, upon mitochondria damage, the accumulated PINK1 (PTEN induced putative kinase 1) can induce autophagy by blocking the release of cytochrome c, which needs further investigations (Boosani et al., 2019).

## Dual Roles of Autophagy in Chemoresistance of Gastric Cancer

**FIGURE 5:**
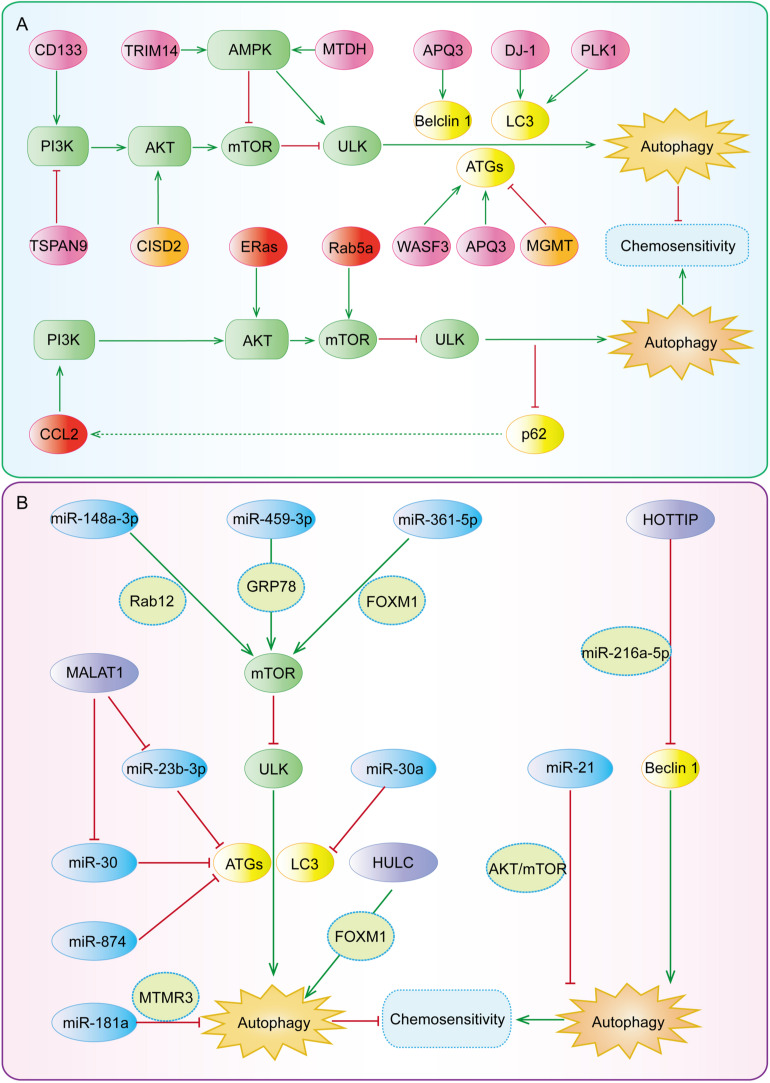
The key regulatory nodes in autophagy-mediated gastric cancer chemoresistance. Various regulatory nodes, including several **(A)** proteins and **(B)** ncRNAs, are involved in the regulation of autophagy, thereby enhancing or reversing the chemotherapy resistance of gastric cancer.

Autophagy functions as a double-edged sword for chemoresistance of gastric cancer. On one hand, autophagy can protect gastric cancer cells from the cytotoxicity of chemotherapy drugs and contribute to the formation of chemoresistance (Russi et al., 2019). On the other hand, it can reverse chemoresistance by promoting apoptosis and/or inhibiting EMT (Nie et al., 2020). The main reason is that autophagy is regulated by multiple proteins and/or ncRNAs and thus plays different roles in gastric cancer chemoresistance (Tsai et al., 2020; Zeng et al., 2020). Therefore, if properly applied, targeting autophagy may be an important strategy for the prevention and treatment of chemoresistance. In this section, we provide a summary of the regulation of autophagy by various proteins (Table 1) and ncRNAs (Table 2) as well as its different roles in gastric cancer chemoresistance (Figure 5).

**TABLE 1:** Key regulatory proteins in autophagy-mediated chemoresistance of gastric cancer.

Protein	Expression in GC	Effects on autophagy	Chemotherapy	Effects on chemosensitivity	Downstream pathways	References
WASF3	NA	Inducing	OXA	Decreasing	ATG12	Nie et al., 2020
PLK1	Up	Inducing	DDP	Decreasing	CDC25C, cyclin B1, LC3-I, LC3-II	Chen et al., 2019
AQP3	Up	Inducing	DDP	Decreasing	Atg5, Beclin-1, P62	Dong et al., 2016
DJ-1	Up	Inducing	EPI	Decreasing	LC3-I, LC3-II	Pan et al., 2018
TSPAN9	Up	Inducing	5-FU	Decreasing	PI3K/AKT/mTOR	Qi et al., 2020
CD133	Up	Inducing	DDP	Decreasing	PI3K/AKT/mTOR	Lu et al., 2019
TRIM14	Up	Inducing	5-FU, OXA	Decreasing	AMPK/mTOR	Xiao et al., 2020
MTDH	Up	Inducing	5-FU	Decreasing	AMPK/ATG5	Pei et al., 2018
ERAS	Up	Inhibiting	DDP	Decreasing	AKT/mTOR	Tian et al., 2019
CCL2	Up	Inhibiting	DDP	Decreasing	PI3K/AKT/mTOR, SQSTM1	Xu et al., 2018b
RAB5A	Up	Inhibiting	DDP	Decreasing	mTOR	Xu et al., 2018a
MGMT	NA	Inhibiting	DDP	Increasing	ATG4B	Lei et al., 2020
CISD2	Down	Inhibiting	5-FU	Increasing	AKT/mTOR	Sun et al., 2017

*5-FU, 5-fluorouracil; DDP, cisplatin; EPI, epirubicin; GC, gastric cancer; OXA, oxaliplatin; NA, not applicable*.

**TABLE 2:** Key regulatory non-coding RNAs in autophagy-mediated chemoresistance of gastric cancer.

Biomarker	Expression in GC	Effects on autophagy	Chemotherapy	Effects on chemosensitivity	Downstream pathways	References
miR-874	Down	Inhibiting	DDP	Increasing	ATG16L1	Huang et al., 2018
miR-30a	Down	Inhibiting	DDP	Increasing	MDR1, P-gp	Du et al., 2018
miR-181a	Down	Inhibiting	DDP	Increasing	MTMR3	Lin et al., 2017
miR-23b-3p	Down	Inhibiting	DDP, VCR, 5-FU	Increasing	ATG12, HMGB2	An et al., 2015
miR-148a-3p	Down	Inhibiting	DDP	Increasing	RAB12, mTORC1	Li et al., 2017
miR-495-3p	Down	Inhibiting	ADM, DDP, 5-FU, VCR	Increasing	GRP78/mTOR	Chen et al., 2018
miR-361-5p	NA	Inhibiting	DOC	Increasing	PI3K/AKT/mTOR, FOXM1	Tian et al., 2018
miR-21	NA	Inhibiting	DDP	Decreasing	PI3K/AKT/mTOR, Beclin-1, LC3	Gu et al., 2020
HOTTIP	Up	Inhibiting	DDP	Decreasing	miR-216a-5p, BCL-2, Beclin-1	Zhao et al., 2020
MALAT1	Up	Inducing	DDP, VCR	Decreasing	miR-30b, ATG5, miR-23b-3p, ATG12	YiRen et al., 2017; Xi et al., 2019
HULC	NA	Inducing	DDP	Decreasing	FoxM1	Xin et al., 2019

*5-FU, 5-fluorouracil; ADM, adriamycin; DDP, cisplatin; DOC, docetaxel; GC, gastric cancer; VCR, vincristine; NA, not applicable.*

### Key Regulatory Proteins in Autophagy-Mediated Gastric Cancer Chemoresistance

Several proteins have shown regulatory effects on autophagy-mediated gastric cancer chemoresistance (Figure 5A) by (i) inducing both autophagy and chemoresistance; (ii) inhibiting autophagy but inducing chemoresistance; or (iii) inhibiting both autophagy and chemoresistance. Based on the modulatory effects of these proteins on autophagy and chemoresistance, developing their inhibitors or activators and combining these compounds with autophagy inhibitors or activators have been emerging as a promising strategy to overcome chemotherapy resistance.

#### Regulatory Proteins Inducing Both Autophagy and Chemoresistance

It has recently been reported that several proteins (Table 1) can induce autophagy by regulating autophagy regulatory genes, such as Beclin-1, ATG12, ATG5, p62, LC3-I, LC3-II, and so on. Nie et al. (2020) have reported that wiskott-aldrich syndrome protein family member 3 (WASF3) is an obstacle for the sensitivity of gastric cancer to oxaliplatin (OXA). It has been found that WASF3 overexpression induces autophagy by increasing ATG12 level and causes OXA resistance in gastric cancer cells, while interference with WASF3 can reverse OXA resistance *in vitro* (Nie et al., 2020). Chen et al. (2019) have demonstrated that the expression of polo-like kinase 1 (PLK1) is significantly increased in cisplatin (DDP)-resistant SGC-7901/DDP cells, whereas PLK1 knockdown inhibits autophagy, increases apoptosis, and restores the chemosensitivity of DDP-resistant cells. Aquaporins (AQPs) are a family of small integral membrane proteins; among them, AQP3 is highly expressed in gastric cancer tissues (Moosavi and Elham, 2020). Dong et al. (2016) have shown that the upregulation of AQP3 increases the expression of ATG5 and Beclin-1, decreases the expression of p62, induces autophagy, and causes DDP resistance, while the application of autophagy inhibitor chloroquine (CQ) can reverse DDP resistance. DJ-1, also known as Parkinson’s disease associated protein 7 (PARK7), is highly expressed in several types of cancer and has been proposed as a chemoresistance-related factor (Jin, 2020). Indeed, DJ-1 is highly expressed in gastric cancer tissues and Epirubicin (EPI)-resistant gastric cancer cells, and EPI treatment can increase the expression of DJ-1 in a dose-dependent manner (Pan et al., 2018). Pan et al. (2018) have reported that the overexpression of DJ-1 significantly increases LC3-II level, induces autophagy, and attenuates EPI-induced apoptosis, while DJ-1 knockdown can reduce autophagy and increase apoptosis, thereby reversing EPI resistance in MGC803 and SGC7901 gastric cancer cells.

Some regulatory proteins have been found to induce autophagy and chemoresistance by regulating the PI3K/AKT/mTOR signaling pathway (Table 1). Tetraspanin 9 (TSPAN9), a member of four transmembrane protein superfamily that plays an important role in tumor progression, has shown regulatory effects on proliferation, migration, invasion, and autophagy (Qi et al., 2019, 2020). Qi et al. (2020) have reported that the protein level of TSPAN9 is increased in 5-fluorouracil (5-FU)-resistant gastric cancer cells, which inhibits the catalytic activity of PI3K through binding to PIK3R3 (p55) and promotes autophagy, whereas knockdown of p55 can enhance the sensitivity of gastric cancer cells to 5-FU. The cluster of differentiation 133 (CD133), an important stemness-related marker has also been related to autophagy (Liu Y. C. et al., 2020b). Lu et al. (2019) have reported that the expression level of CD133 and the percentage of CD133-positive cells are increased in DDP-resistant gastric cancer cells, promoting cancer stem cell (CSC) properties of DDP-resistant cells. Further studies have shown that CD133 can increase autophagy and the stemness of gastric cancer cells via activating the PI3K/AKT/mTOR signaling pathway, causing DDP resistance of gastric cancer cells *in vivo* and *in vitro* (Lu et al., 2019).

The regulatory proteins of the AMPK pathway have been shown to induce autophagy and chemoresistance (Table 1). Xiao et al. (2020) have suggested that tripartite motif containing 14 (TRIM14) is significantly up-regulated in 5-FU- and OXA-resistant gastric cancer tissues and cell lines. Further studies have shown that TRIM14 can promote autophagy and induce chemoresistance by activating the AMPK/mTOR pathway *in vivo* and *in vitro* while silencing TRIM14 provides the opposite effects (Xiao et al., 2020). Pei et al. (2018) have found that metadherin (MTDH) expression in gastric cancer tissues is significantly increased and positively correlated with 5-FU resistance. Further mechanism studies have suggested that MTDH can promote AMPK phosphorylation, upregulate ATG5 expression, activate autophagy, and consequently induce 5-FU resistance (Pei et al., 2018).

#### Regulatory Proteins Inhibiting Autophagy but Inducing Chemoresistance

Embryonic stem cell-expressed Ras (ERas) is a novel member of the Ras protein family and is highly expressed in gastric, colorectal, and breast cancer (Suárez-Cabrera et al., 2018; Tian et al., 2019). Tian et al. (2019) have found that ERas overexpression significantly increases the phosphorylation level of mTOR (Ser2448) and its substrate (ULK1-Ser757), as well as AKT-Ser473, activates the AKT/mTOR pathway, blocks autophagic flux in gastric cancer cells, suppresses DDP-induced apoptosis, and induces DDP resistance. In contrast, silencing ERas has been found to increase autophagic flux and enhance the sensitivity of gastric cancer cells to DDP *in vitro* (Tian et al., 2019).

Chemokine C-C motif ligand 2 (CCL2), is a well-known cytokine belonging to the CC chemokine family 9, which can affect drug sensitivity in both paracrine and autocrine manners (Hao et al., 2020). CCL2 expression has been related to tumor invasion, metastasis, and drug resistance (Zhong et al., 2018; Dutta et al., 2020; Hao et al., 2020). Xu et al. (2018b) have found that DPP-resistant gastric cancer cell lines, such as BGC823/DDP cells and SGC7901/DDP cells secrete more CCL2 to maintain DDP resistance. It has further been found that CCL2 overexpression increases the expression of p62 by activating PI3K/AKT/mTOR signaling pathway, whilst the increased expression of p62, in turn, activates the transcription of CCL2, inhibits autophagy, and forms a positive feedback loop to maintain drug resistance (Xu et al., 2018b).

Rab5a, also known as RAB5, is a member of the Rab family of small GTPases and is involved in the autophagy process by blocking autophagosome-lysosome fusion (Lu et al., 2014). Xu et al. (2018a) have shown that Rab5a expression level is positively associated with drug resistance in gastric cancer cells. It has been found that Rab5a overexpression increases the phosphorylation level of mTOR, decreases the LC3-II/I ratio, and increases p62 expression level, thereby inhibiting autophagy and inducing DDP resistance, whereas Rab5a knockdown can facilitate autophagy and reverse DDP resistance *in vitro* (Xu et al., 2018a).

#### Regulatory Proteins Inhibiting Both Autophagy and Chemoresistance

*O*-6-methylguanine-DNA methyltransferase (MGMT) is a type of suicide DNA damage repair enzymes, and DNA damage repair disorders have been reported to induce autophagy (Balvers et al., 2015; Abdu et al., 2020). Lei et al. (2020) have found that the high expression of MGMT is significantly correlated with the low expression of ATG4B and the favorable prognosis of gastric cancer. DDP can inhibit MGMT expression in a dose- and time-dependent manner and the low expression of MGMT can, in turn, induce autophagy and cisplatin resistance. Overexpression of MGMT can inhibit autophagy and reverse DDP resistance *in vivo* and *in vitro* (Lei et al., 2020).

CDGSH iron sulfur domain 2 (CISD2) is a survival gene and plays an important role in the redox reaction, longevity, tumorigenesis, and tumor progression (Li et al., 2019; Nobili et al., 2020). Sun et al. (2017) have shown that CISD2 is significantly down-regulated in gastric cancer tissues and drug-resistant cell lines, which contributes to the resistance of gastric cancer cells to 5-FU. Further studies have found that CISD2 overexpression antagonizes 5-FU-induced autophagy by increasing the phosphorylation levels of AKT (S473) and mTOR (Ser2448), consequently reversing the reduction of p-MTOR and p-AKT by 5-FU and enhancing the sensitivity of MKN1 and BGC823 cells to 5-FU (Sun et al., 2017).

### Regulatory Effects of miRNAs on Autophagy-Mediated Gastric Cancer Chemoresistance

MiRNAs, as a kind of highly conserved short ncRNAs, have a length of 18–25 nucleotides, regulate the expression of many genes, and participate in the development of human cancer and drug resistance (Poursheikhani et al., 2020; Ratti et al., 2020). miRNA-mediated autophagy (Figure 5B) has been found to play a role in chemoresistance in gastric cancer by regulating autophagy-related genes or signaling pathways (Jamali et al., 2020).

To date, many miRNAs, including miR-874, miR-30, miR-181a, miR-23b-3p, miR-148a-3p, miR-495-3p, miR-361-5p, and miR-21 have been involved in the regulation of autophagy and chemoresistance in gastric cancer (as summarized in Table 2). Among them, the expression of miR-874, miR-30, miR-181a, and miR-23b-3p are down-regulated in drug-resistant gastric cancer cell lines and gastric cancer tissues (An et al., 2015; Lin et al., 2017; Du et al., 2018; Huang et al., 2018). Huang et al. (2018) have found that miR-874 inhibits autophagy by down-regulating ATG16L1, thus enhancing the sensitivity of gastric cancer cells to DDP *in vitro* and *in vivo*. Du et al. (2018) have reported that miR-30a up-regulates the expression of P-gp and MDR1, decreases the LC3-II/I ratio, inhibits autophagy, and reverses DDP resistance. Lin et al. (2017) have shown that miR-181a directly targets the 3′-UTR of myotubularin related protein 3 (MTMR3), decreases the mRNA and protein levels of MTMR3, inhibits autophagy, and increases the sensitivity of gastric cancer cells to DDP. An et al. (2015) have demonstrated that miR-23b-3p directly targets ATG12 and high-mobility group box 2 (HMGB2), inhibits autophagy, and increases the sensitivity of gastric cancer cells to DDP, vincristine (VCR), and 5-FU.

It has been found that both miR-148a-3p and miR-495-3p are lowly expressed in drug-resistant gastric cancer cell lines and tumor tissues (Li et al., 2017; Chen et al., 2018). Li et al. (2017) have found that miR-148a-3p down-regulates the expression of A-kinase anchoring protein 1 (AKAP1) and RAB12 (RAB12, member RAS oncogene family) and reduces the inhibitory effects of RAB12 on mTORC1, thereby inhibiting autophagy and reversing the resistance of gastric cancer cells to DDP. Chen et al. (2018) have reported that miR-495-3p directly targets glucose regulated protein 78 (GRP78) at the post-transcriptional level, activates mTOR signaling, inhibits autophagy, and reverses multidrug resistance (MDR) in gastric cancer. Tian et al. (2018) have suggested that miR-361-5p negatively regulates the expression of FOXM1, which further causes an increase in the expression of p-AKT and mTOR, the inhibition of autophagy, and the enhanced sensitivity of gastric cancer cells to Docetaxel (DOC) *in vitro*. Gu et al. (2020) have found that miR-21 overexpression can increase the phosphorylation of AKT and mTOR, inhibit autophagy, and induce DDP resistance. It has been further demonstrated that the knockdown of miR-21 can promote autophagy and then sensitize DDP-resistant gastric cancer cell lines to DDP (Gu et al., 2020).

### Regulatory Effects of lncRNAs on Autophagy-Mediated Gastric Cancer Chemoresistance

LncRNAs are the transcripts of more than 200 nucleotides, accounting for 80–90% of all ncRNAs, and regulate gene expression at the pre-transcriptional, transcriptional, and post-transcriptional levels (Yuan et al., 2020). Therefore, lncRNAs widely participate in various physiological and pathological processes of organisms (Yuan et al., 2020). LncRNAs have been found to regulate autophagy and drug resistance (Figure 5B) by acting as a competing endogenous RNA (ceRNA) or directly binding to proteins to modulate their expression and functions (as summarized in Table 2).

It has been found that the expression levels of HOTTIP (HOXA distal transcript antisense RNA), MALAT1 (metastasis associated lung adenocarcinoma transcript 1), and HULC (hepatocellular carcinoma up-regulated long non-coding RNA) are elevated in chemotherapy-resistant gastric cancer cell lines and tumor tissues (YiRen et al., 2017; Xin et al., 2019; Zhao et al., 2020). Zhao et al. (2020) have indicated that HOTTIP may function as a ceRNA of miR-216a-5p, increasing the expression level of BCL-2 and decreasing the expression level of Beclin-1. Further studies have shown that HOTTIP overexpression inhibits autophagy and induces DDP resistance in gastric cancer while the silencing of HOTTIP increases the sensitivity of gastric cancer cells to DDP (Zhao et al., 2020). YiRen et al. (2017) have reported that MALAT1 acts as a ceRNA against miR-23b-3p and attenuates the inhibitory effects of miR-23b-3p on ATG12, leading to autophagy induction and VCR resistance in gastric cancer cells. Moreover, Xi et al. (2019) have found that MALAT1 can competitively bind to miR-30 and promote the expression of ATG5, thus inducing autophagy and DDP resistance. Xin et al. (2019) have shown that HULC can stabilize FoxM1 by inhibiting its ubiquitination and induce autophagy and DDP resistance. It has further been observed that intervening HULC can suppress autophagy and then reduce the resistance of gastric cancer cells to DDP *in vitro* (Xin et al., 2019).

## Modulatory Effects of Autophagy Inhibitors and Activators on Gastric Cancer Chemoresistance

Given the importance of autophagy in chemotherapy resistance, the development of autophagy inhibitors or activators may provide a new opportunity for the treatment of human cancer, especially for those with drug resistance (Mele et al., 2020). FDA has approved the clinical application of CQ and its derivative hydroxychloroquine (HCQ), which can inhibit autophagy by blocking autophagosome fusion and degradation (Liu T. et al., 2020a). At present, there are several clinical studies on the treatment of multiple tumors with HCQ alone or in combination with chemotherapy. Various natural products from TM, medicinal plants, or microorganisms, such as flavonoids, alkaloids, terpenoids, coumarins, etc. have been reported as potential autophagy inhibitors and activators and MDR reversal agents (as summarized in Table 3) (Sato et al., 2019; Liu S. et al., 2020; Nazim et al., 2020).

**TABLE 3:** Modulatory effects of autophagy inhibitor and activators on gastric cancer chemoresistance.

Compounds	Source	Effects on autophagy	Chemotherapy	Effects on chemosensitivity	Downstream pathways	References
Genipin	Natural products from TM	Inducing	OXA	Increasing	p53, DRAM	Kim et al., 2020
Tanshinone IIA	Natural products from TM	Inducing	ADM	Increasing	LC3-II, P62	Xu Z. et al., 2018
Liquiritin	Natural products from TM	Inducing	DDP	Increasing	LC3B, Beclin-1, p62	Wei et al., 2017
DSGOST	Prescription from TM	Inducing	DDP	Increasing	LC3-II, ATG5, Beclin-1, Bcl2, AMPKα/ULK1	Kim T. W. et al., 2018
Cucurbitacin B	Natural products from edible plants	Inducing	DDP	Increasing	CIP2A/PP2A/mTORC1	Dandawate et al., 2020
Phloretin	Natural products from edible plants	Inducing	ADM	Increasing	ERK1/2, MAP, LC3B II, Beclin-1	You et al., 2020
Tunicamycin	Antibiotic	Inducing	ADM, VCR	Increasing	LC3-I, LC3-II	Wu et al., 2018
Indomethacin	NSAIDs	Inhibiting	OXA	Increasing	LC3-II, LC3-II, p62, NBR1	Vallecillo-Hernandez et al., 2018
Propofol	Anesthetic	Inhibiting	DDP	Increasing	MALAT1, miR-30e, ATG5	Zhang Y.F. et al., 2020

*ADM, adriamycin; DDP, cisplatin; OXA, oxaliplatin; TM, traditional medicine; VCR, vincristine*.

### Effects of Autophagy Activators on Gastric Cancer Chemoresistance

Genipin, a natural product derived from traditional Chinese medicine (TCM) *Gardenia jasminoides*, has shown anti-angiogenic, anti-proliferative, anti-inflammatory, and anticancer activities (Jo et al., 2019; Zhang C. et al., 2020; Zhou et al., 2020). Kim et al. (2020) have found that genipin can increase the expression of p53 and DRAM, induce apoptosis and autophagy, and enhance the sensitivity of AGS and MKN45 gastric cancer cell lines to OXA. Tanshinones are a group of abietane diterpenes from the TCM Danshen and have exhibited anticancer activities *in vitro* and *in vivo* (Li M. et al., 2020). Xu Z. et al. (2018) have reported that tanshinone IIA can reduce the expression of multidrug resistance protein 1 (MRP1) and inhibit the efflux of adriamycin (ADM). Besides, the combination of tanshinone IIA with ADM can induce autophagy, thus promoting cell apoptosis and enhancing the sensitivity of gastric cancer cells to ADM (Xu Z. et al., 2018). Liquiritin is the main medicinal component of TCM licorice. Wei et al. (2017) have found that liquiritin increases Beclin-1 expression, inhibits p62 expression, triggers autophagy and apoptosis, and then reverses DDP resistance *in vitro* and *in vivo*.

Danggui-Sayuk-Ga-Osuyu-Saenggang-Tang (DSGOST) is a traditional Korean herbal medicine (Kim T. W. et al., 2018). DSGOST is similar to TCM Danggui-Sini-Jia-Wuzhuyu-Shengjian-Tang and is commonly used in treating patients with Raynaud’s phenomenon caused by multiple chemotherapy drugs (Kim T. W. et al., 2018). Kim T. W. et al. (2018) have reported that DSGOST can cause the dissociation of the Beclin-1-Bcl2 complex, activate the AMPK/ULK1 pathway, increase the autophagy flux, induce autophagy and apoptosis, and enhance the sensitivity of gastric cancer cell lines AGS and SNU-638 to DDP.

The dietary natural products, cucurbitacin B and phloretin have recently been found to exert anticancer activities and reverse chemotherapy resistance (Hsiao et al., 2019; Dandawate et al., 2020). Xu et al. (2020) have shown that cucurbitacin B induces autophagy and apoptosis and reverses the sensitivity of DDP-resistant cells to DDP. The mechanisms of action studies have indicated that cucurbitacin B inhibits CIP2A (cancerous inhibitor of protein phosphatase 2A), subsequently reactivating PP2A (protein phosphatase 2A) and enhancing PP2A-dependent mTORC1 inactivation (Xu et al., 2020). You et al. (2020) have found that phloretin inhibits the phosphorylation of ERK1/2 and MAPK p38 and increases the expression of LC3B II and Beclin-1, thus inducing autophagy and enhancing the sensitivity of gastric cancer cells to ADM *in vitro*.

Abnormal glycosylation has been widely regarded as an important sign of cancer and is significantly associated with tumor development, progression, metastasis, and chemoresistance (Liu et al., 2018; Liu T. et al., 2020a). As an effective glycosylation inhibitor, tunicamycin has been initially identified as a natural antibiotic and anticancer compound (Liu Y. C. et al., 2020b; Santos-Laso et al., 2020). Wu et al. (2018) have suggested that tunicamycin inhibits *N*-glycosylation to aggravate ER stress, induces autophagy, and increases the sensitivity of gastric cancer cells to ADM and VCR.

### Effects of Autophagy Inhibitors on Gastric Cancer Chemoresistance

Indomethacin, a common non-steroidal anti-inflammatory drug (NSAID), has been reported as a coadjutant of anticancer drugs with satisfactory efficacy (López-Contreras et al., 2019; Seetha et al., 2020). Vallecillo-Hernandez et al. (2018) have found that indomethacin can induce the accumulation of p62 and neighbor of BRCA1 (NBR1), impair lysosomal function, inhibit autophagic degradation, and increase OXA-induced cell death in AGS cells. Propofol, a sedative widely used in surgery, has shown efficacy in several types of cancer, such as pancreatic cancer (Wang et al., 2020), gastric cancer (Liu F. et al., 2020), colon cancer (Liu F. et al., 2020), papillary thyroid carcinoma (Li Y. et al., 2020), and so on. Zhang et al. have demonstrated that propofol in combination with DDP inhibits the expression of lncRNA MALAT1 and enhances the inhibitory effects of miR-30e on ATG5 and autophagy, thereby increasing the sensitivity of gastric cancer cells to DDP *in vivo* and *in vitro* (Zhang Y.F. et al., 2020).

## Conclusion and Future Direction

Overall, this review provides compelling evidence for the dual roles of autophagy in the chemoresistance of gastric cancer. Autophagy can protect cancer cells from chemotherapy and participate in the formation of MDR, while it can also promote apoptosis and kill MDR cancer cells. Therefore, the development of autophagy inhibitors or activators may be an important way to reverse drug resistance and enhance chemosensitivity. The combination of autophagy modulators and chemotherapy drugs will also bring new hope for the treatment of human cancer. For instance, it is exciting to note that the combination of autophagic inhibitor HCQ and gemcitabine or nab-paclitaxel can significantly improve the overall response rate of cancer patients (Karasic et al., 2019).

The dual roles of autophagy in drug resistance are mainly regulated by different proteins and ncRNAs. Proteins and ncRNAs can monitor autophagy by regulating autophagy regulatory proteins and/or autophagy regulatory signaling pathways. In the fields of drug resistance and autophagy in gastric cancer, researchers have focused on PI3K/AKT and MAPK pathways. More studies on other autophagy-related pathways (p53, MAPK, or PTEN pathways) are expected in the future. The development of small-molecule inhibitors or activators targeting key regulatory nodes in the process of autophagy may provide an alternative treatment for patients with cancer. For example, SBI-0206965, as a small molecule inhibitor of ULK1, has been found to enhance daunorubicin sensitivity in acute myeloid leukemia (Qiu et al., 2020). Besides, the ULK1 activator LYN-1604, which can induce autophagy-related cell death through the ULK complex, shows significant anticancer activity in triple-negative breast cancer (Ouyang et al., 2017). Therefore, further strengthening the research on the role of different proteins and ncRNAs in regulating autophagy-mediated gastric cancer chemoresistance will be beneficial to the development of promising drugs for gastric cancer therapy.

Many natural products from TM and edible plants have shown preventive and therapeutic efficacy in human cancer by targeting multiple signaling pathways and inducing cell cycle arrest and apoptosis (Koh et al., 2020). Accumulating evidence has demonstrated the modulatory effects of these anticancer natural products on autophagy and chemosensitivity. Thus, using natural products alone or in combination with autophagy modulators and/or chemotherapy drugs may exhibit promising efficacy in human cancer, especially in drug-resistant cancer. Nevertheless, further studies are warranted to identify the specific molecular targets of these natural products and examine the efficacy and safety of these strategies in clinically relevant cancer models.

## Author Contributions

J-JQ and X-DC conceptualized the manuscript. J-LX, LY, Y-CT, Z-YX, and H-DX collected the literature, wrote the manuscript, and made the figures. J-JQ edited and made significant revisions to the manuscript. All authors read and approved the final manuscript.

## Conflict of Interest

The authors declare that the research was conducted in the absence of any commercial or financial relationships that could be construed as a potential conflict of interest.
